# Ultrasound-Guided Distal Thoracolumbar Fascia Hydrodissection at the Gluteal Junction: A Technical Report With Cadaveric Assessment of Compartmental Spread and Gross Neural Sparing

**DOI:** 10.7759/cureus.117256

**Published:** 2026-09-30

**Authors:** King Hei Stanley Lam, Yonghyun Yoon, Teinny Suryadi, Rowook Park, Daniel Chiung-Jui Su

**Affiliations:** 1 Clinical Research, International Academy of Regenerative Medicine, Incheon, KOR; 2 The Board of Clinical Research, The Indonesian Malaysian Regenerative Institute, Jakarta, IDN; 3 The Board of Clinical Research, The International Association of Musculoskeletal Medicine, Kowloon, HKG; 4 Faculty of Medicine, The Chinese University of Hong Kong, New Territories, HKG; 5 Faculty of Medicine, The University of Hong Kong, Hong Kong, HKG; 6 The Board of Clinical Research, The Hong Kong Institute of Musculoskeletal Medicine, Kowloon, HKG; 7 Orthopedics, International Academy of Musculoskeletal Medicine, Hong Kong, HKG; 8 Orthopedics, International Academy of Regenerative Medicine, Incheon, KOR; 9 Orthopedic Surgery, Hallym University Kangnam Sacred Heart Hospital, Seoul, KOR; 10 Orthopedic Surgery, Incheon Terminal Orthopedic Surgery Clinic, Incheon, KOR; 11 Physical Medicine and Rehabilitation, Medistra Hospital, Jakarta, IDN; 12 Physical Medicine and Rehabilitation, Synergy Clinic, Jakarta, IDN; 13 Physical Medicine and Rehabilitation, Hermina Podomoro Hospital, Jakarta, IDN; 14 Rehabilitation Medicine, Sae Yonsei Rehabilitation Clinic, Seoul, KOR; 15 Physical Medicine and Rehabilitation, Chi Mei Medical Center, Tainan, TWN

**Keywords:** cadaveric study, chronic low back pain, gluteus maximus, hydrodissection, longissimus thoracis, multifidus, radicular-like pain, sacrotuberous ligament, thoracolumbar fascia, ultrasound-guided injection

## Abstract

Chronic low back pain with radiating lower-extremity symptoms in the absence of clear radiculopathy or major central canal stenosis remains a common diagnostic and therapeutic challenge. In patients with radiating low back pain without a convincing compressive correlate, posterior myofascial structures may be considered among several potential contributors; however, the present report evaluates the anatomical feasibility of a distal thoracolumbar fascia (TLF) hydrodissection approach rather than a clinical pain generator. We developed an ultrasound-guided hydrodissection technique targeting the distal TLF deep to the gluteus maximus and inferior to the sacroiliac joint. This technical report describes the clinical rationale, procedural technique, and cadaveric dye-spread assessment of injectate distribution and gross neural sparing.

The technique was developed for a selected clinical context characterized by chronic low back pain radiating to the posterior thigh or calf, intact motor function, and lumbar magnetic resonance imaging (MRI) findings that may include fatty infiltration of the lower lumbar multifidus, with or without involvement of the gluteal origin region, after limited or unsustained benefit from standard image-guided interventions. For the described clinical technique, the symptomatic side would receive 10 mL of injectate prepared to a final concentration of 15% dextrose with 0.1% lignocaine. For cadaveric assessment, three fresh-frozen cadavers (two male, one female; mean age 74.3 years) underwent bilateral ultrasound-guided injection of the same target plane, yielding six injected sides. Each side received 10 mL of undiluted 1% methylene blue. Layered dissection demonstrated cephalad spread to approximately the L3 level in six out of six sides (100%). Multifidus staining occurred in six out of six (100%) and longissimus thoracis staining in six out of six (100%), whereas iliocostalis lumborum staining occurred in 0 out of six (0%). Partial proximal sacrotuberous ligament staining was observed in six out of six (100%). No gross dye was observed in the sciatic nerve, sacral plexus, neural foramina, or epidural space in 0 out of six sides (0%).

In this fresh-frozen cadaver model, ultrasound-guided injection at the distal TLF-gluteal junction was technically achievable and produced a consistent gross cephalad dye-spread pattern toward the posterior fascial-paraspinal region. Because both sides of each cadaver were injected, the present design was not intended to determine contralateral crossover. These cadaveric findings do not exclude microscopic spread and do not establish histologic or in vivo safety. They also do not establish safety or efficacy of the described injectate formulation at this target in living patients. This report should be interpreted as hypothesis-generating rather than evidence of clinical effectiveness or safety.

## Introduction

Low back pain with radiation into the lower limb is often approached as a spinal or radicular problem. In many patients, that is appropriate. However, there is a smaller group in whom the history suggests sciatica-like pain, but the examination and imaging do not convincingly support nerve root compression. These patients may report pain extending into the buttock, posterior thigh, or calf, yet motor function remains intact, sensory findings are minimal, and lumbar MRI shows only mild degenerative change. Standard image-guided procedures such as facet injections, sacroiliac joint injections, and selective nerve root blocks may then provide little benefit or only transient relief.

The thoracolumbar fascia (TLF) has increasingly been recognized as a biologically active structure with important mechanical and nociceptive roles in low back pain [1-6]. Anatomically, the TLF is a multilayered fascial complex that invests the paraspinal musculature, transmits tensile forces across muscular attachments, and forms distinct compartmental relationships with the multifidus, longissimus, and iliocostalis musculature [1-3,7]. MRI-defined fatty infiltration of the lumbar multifidus has been associated with chronic low back pain and impaired lumbar function [8-10]. The proximal sacrotuberous ligament, which forms part of the posterior pelvic soft-tissue continuum, may also be relevant in this region [11]. Histologic and experimental data further suggest that the TLF contains dense sensory innervation, including nociceptive fibers, and may evoke more intense and more widely radiating pain than adjacent muscle when chemically stimulated [4-6,12,13]. Although these findings support the hypothesis that thoracolumbar fascial structures may contribute to symptoms in selected clinical contexts, that hypothesis is not tested directly in the present cadaveric study.

Previous anatomical studies have described TLF anatomy and related paraspinal compartmental relationships, but to our knowledge no published cadaveric study has specifically evaluated injectate spread from the distal gluteal thoracolumbar fascial junction under ultrasound guidance. The integration of imaging services into primary care has also been discussed as a broader healthcare-delivery priority, with potential to support earlier assessment and more accessible image-guided management pathways [14]. Within this context, the present report examines the anatomical feasibility of a specific ultrasound-guided distal TLF hydrodissection approach.

On the basis of these clinical and anatomical observations, we developed an ultrasound-guided hydrodissection technique aimed at the distal TLF where it meets the deep gluteal fascial plane below the sacroiliac joint. The target was chosen as a hypothesized caudal sonographic entry point into the deep posterior fascial-paraspinal region rather than as a previously validated spread pathway.

Accordingly, the objectives of this technical report were to (1) describe the ultrasound-guided distal TLF hydrodissection technique, (2) document cadaveric dye spread following injection, and (3) assess gross neural sparing on layered dissection.

## Technical report

Clinical target phenotype

The following clinical target phenotype is presented to illustrate the intended clinical application of the technique rather than to describe participants enrolled or systematically analyzed in this cadaveric study. The intended clinical context for this technique assumes prior assessment for serious underlying pathology and progressive neurological deficits, rather than relying solely on the absence of a clear compressive correlate on MRI [15].

In patients presenting with radiating low back pain in the absence of a convincing compressive correlate, posterior myofascial structures may be considered among several potential contributors. The present cadaveric technical report was designed to evaluate the anatomical feasibility of a distal TLF hydrodissection approach rather than to establish a clinical pain generator.

The typical clinical context in which this technique may be considered includes chronic low back pain of more than three months’ duration with radiation into the posterior thigh or calf. Radiation to the plantar sole is not a usual feature. On examination, motor power is expected to be normal. Sensory examination may be normal or show only minor change, such as a mild reduction in pinprick sensation in a limited dermatomal distribution. Lumbar MRI may show mild spondylosis, disc desiccation, small central disc protrusion, or mild foraminal narrowing, but without a structural lesion that clearly explains the severity or distribution of symptoms. MRI may also demonstrate fatty infiltration involving the lower lumbar multifidus, with or without involvement of the gluteal origin region. Within the scope of this technical report, fatty infiltration refers to routine clinical imaging impression only; no formal grading system or study-specific MRI reading protocol was applied.

In the authors’ clinical practice, this technique was considered in selected patients after limited or unsustained benefit from other image-guided interventions, including lumbar facet injections, sacroiliac joint injections, and selective nerve root blocks. This sequence is presented as practice context only and should not be interpreted as a standardized or evidence-based treatment algorithm. It was also considered in selected patients with persistent symptoms despite prior lumbar decompression surgery performed elsewhere.

A summary of typical selection features is shown in Table 1.

**Table 1:** Proposed clinical selection features for distal thoracolumbar fascia hydrodissection Summary of the intended clinical context in which this ultrasound-guided distal thoracolumbar fascia hydrodissection technique may be considered, including symptom pattern, neurological findings, MRI characteristics, prior treatment context, and procedural rationale. This table does not describe a formally enrolled clinical cohort.

Domain	Proposed feature
Pain duration	Chronic low back pain >3 months
Pain radiation	Posterior buttock/thigh, mid-thigh, or distal calf
Pain distribution exclusion	No radiation to plantar sole
Motor examination	Normal motor function
Sensory examination	Normal or only mild sensory change
MRI structural findings	Mild spondylosis, disc desiccation, small central disc prolapse, or mild foraminal narrowing without convincing compressive correlate
MRI soft-tissue findings	Fatty infiltration of lower lumbar multifidus with or without involvement of the gluteal origin region
Prior treatment response	Inadequate or transient response to facet, sacroiliac joint, and selective nerve root injections
Intended procedural context	Posterior myofascial/fascial structures considered among several possible contributors, without implying a validated diagnostic pain source

Anatomical rationale

The anatomical rationale for this technique is that the distal TLF at the gluteal junction may provide a caudal sonographic entry point into a deep posterior fascial-paraspinal region. In the present cadaveric model, dye spread was observed cephalad toward the multifidus and longissimus thoracis compartments, but this should not be interpreted as proving a discrete universal anatomical channel [1-3,7]. This anatomical rationale informed target selection, whereas the actual spread pattern observed in the present study was assessed subsequently through cadaveric dye dissection.

The target lies inferior to the sacroiliac joint and deep to the gluteus maximus, where the distal TLF can be approached under ultrasound guidance as a distinct interfascial plane. The iliocostalis lumborum was hypothesized a priori to lie outside the principal spread field because it occupies a more lateral paraspinal compartment relative to the medial/intermediate fascial pathway targeted in the present technique. Although the anatomical relevance of the multifidus is supported by prior literature linking paraspinal dysfunction and fatty infiltration with chronic low back pain phenotypes [8-10], the present study does not test whether this region is a clinical pain generator.

Ethical oversight and cadaver specimens

This cadaveric study used donated fresh-frozen human specimens. Ethical approval was obtained from the Innovation Academic Center (approval no. 79-68). All procedures were conducted in accordance with institutional requirements for research involving donated human tissue.

Three fresh-frozen cadavers were included (two male, one female; mean age 74.3 years). This represented a convenience sample based on specimen availability for an exploratory anatomical feasibility study. Bilateral injections were performed in each cadaver, yielding six injected sides for descriptive side-level reporting. The cadavers constituted the biological specimens, whereas injected sides were treated as descriptive observational units rather than independent inferential samples. Cadavers were included if the lumbosacral, gluteal, and proximal posterior thigh regions were intact and suitable for ultrasound-guided injection and subsequent dissection. Cadavers were excluded if they had prior lumbosacral or gluteal surgery, major regional deformity, extensive soft tissue disruption, or gross pathology that would interfere with injection-pathway interpretation.

Cause of death and relevant comorbidities were not available from donation program records. Cadavers were stored at 2-4°C and had undergone one freeze-thaw cycle. The interval from death to freezing was within 24 hours, and specimens were thawed according to institutional protocol for three hours before injection. No prior dissection of the target region had been performed before injection.

Operator details

All injections were performed under real-time ultrasound guidance by a single operator with 20 years of experience in musculoskeletal ultrasound-guided interventions and more than 10,000 prior fascial or interfascial hydrodissection procedures before this study. The same operator performed all cadaveric injections. Cadaveric dissections were performed by a separate anatomist. The anatomist was aware that bilateral injections had been performed and therefore was not blinded to the possibility of dye on either side, nor to side or injection sequence during dissection. However, the anatomist was blinded to the authors’ expected spread pattern, expected cranial extent of spread, expected ligament involvement, and expected gross neural-sparing findings at the time of interpretation.

Ultrasound equipment and landmark identification

Cadaveric injections were performed under ultrasound guidance using a GE Venue Fit system with an L4-20t linear transducer (General Electric, Boston, MA, USA). The transducer frequency range was 3.4-12.6 MHz, and typical imaging settings included a depth of approximately 3.5 cm, a focal zone at approximately 1.5 cm, and gain adjusted as needed to optimize visualization of the gluteal fascia, gluteus maximus, and distal TLF plane. Depth, gain, and focal settings were adjusted in real time according to tissue depth and body habitus; no formal BMI-based analysis was performed. Color Doppler was used selectively when vascular clarification was required.

The ultrasound examination began at the distal sacral-gluteal region and progressed laterally to identify the inferior margin of the sacroiliac joint, the overlying gluteus maximus, and the fascial plane deep to the gluteus maximus and superficial to the distal TLF. The preferred target view was the level at which the gluteal-TLF junction could be visualized clearly with a safe in-plane needle trajectory that remained inferior to the sacroiliac joint and away from deeper neural structures. Representative landmark identification and procedural setup are shown in Figures 1, 2 and Video 1.

**Figure 1:**
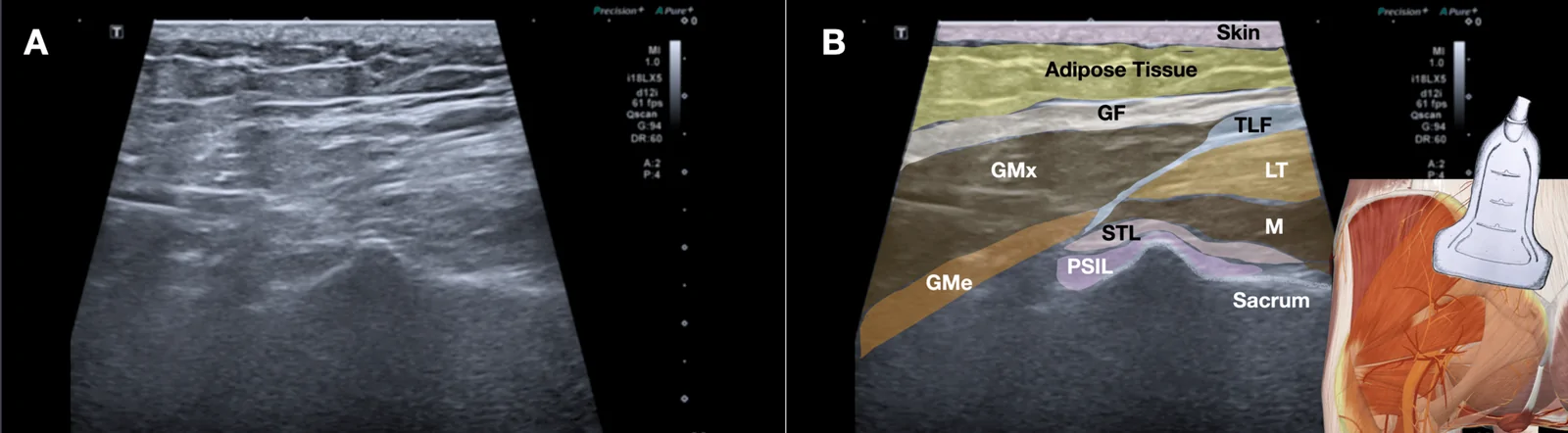
Ultrasound probe position and sonoanatomy at the distal sacral-gluteal junction. (A) Original unlabelled B-mode ultrasound image demonstrating the sonoanatomy at the distal sacral-gluteal junction before injection. (B) Corresponding annotated ultrasound image with colour overlay and labels identifying the relevant sonoanatomy, together with a schematic drawing of the scanned structures and probe position. The target plane lies inferior to the sacroiliac joint, at the deep surface of the gluteus maximus and superficial to the distal thoracolumbar fascia. Abbreviations: GF, gluteal fascia; GMe, gluteus medius; GMx, gluteus maximus; LT, longissimus thoracis; M, multifidus; PSIL, posterior sacroiliac ligament; STL, sacrotuberous ligament; TLF, thoracolumbar fascia. Credit: Schematic diagram created in Keynote (Apple Inc., Cupertino, CA, USA) by Professor King Hei Stanley Lam.

**Figure 2:**
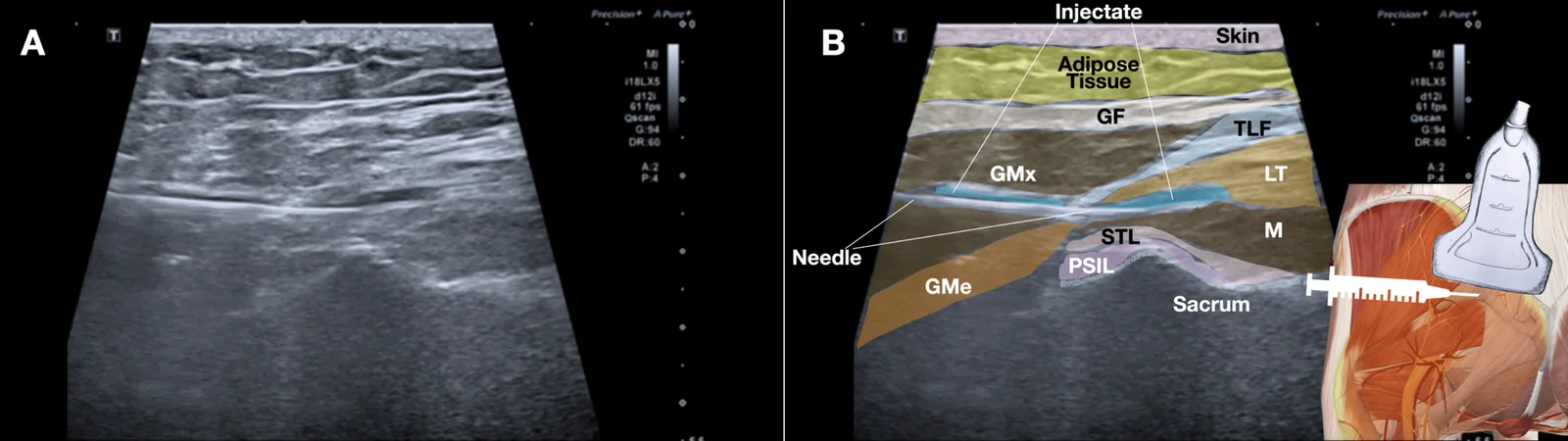
In-plane needle approach to the distal thoracolumbar fascia at the sacral-gluteal junction. (A) Original unlabelled B-mode ultrasound image obtained during injection, showing lateral-to-medial in-plane advancement of a 22-gauge needle into the intended hydrodissection plane at the deep surface of the gluteus maximus and superficial to the distal thoracolumbar fascia at the gluteal junction. (B) Corresponding annotated ultrasound image with colour overlay and labels identifying the relevant sonoanatomy, needle trajectory, and injectate. Abbreviations: GF, gluteal fascia; GMe, gluteus medius; GMx, gluteus maximus; LT, longissimus thoracis; M, multifidus; PSIL, posterior sacroiliac ligament; STL, sacrotuberous ligament; TLF, thoracolumbar fascia. Credit: Schematic diagram created in Keynote (Apple Inc., Cupertino, CA, USA) by Professor King Hei Stanley Lam. Image courtesy of Professor King Hei Stanley Lam.

**Video 1:** Ultrasound-guided distal thoracolumbar fascia hydrodissection at the gluteal junction. This video demonstrates patient positioning, ultrasound probe placement, sonographic identification of the distal sacral landmarks and inferior border of the sacroiliac joint, and the in-plane lateral-to-medial needle approach to the target plane deep to the gluteus maximus and superficial to the distal thoracolumbar fascia. Still images corresponding to Figure 1 and Figure 2 are embedded within the video to highlight key anatomical landmarks and final needle position during hydrodissection. Abbreviations: GF, gluteal fascia; GMe, gluteus medius; GMx, gluteus maximus; LT, longissimus thoracis; M, multifidus; PSIL, posterior sacroiliac ligament; STL, sacrotuberous ligament; TLF, thoracolumbar fascia. Video courtesy of Professor King Hei Stanley Lam.

Step-by-step injection technique

Step 1: Patient Positioning

The patient is placed prone, with a pillow or abdominal support used as needed to reduce lumbar lordosis and improve transducer contact over the distal sacral-gluteal region.

Step 2: Landmark Identification

The linear transducer is positioned over the gluteal junction to identify the inferior border of the sacroiliac joint, the gluteus maximus, and the distal TLF plane. The intended target is the interfascial plane at the deep surface of the gluteus maximus and superficial to the distal TLF.

Step 3: Needle Approach

After skin preparation using standard sterile technique, a 22-gauge 3.5-inch spinal needle or a 22-gauge 4-inch hypodermic needle is advanced in plane from lateral to medial. Although no single fixed numeric insertion angle was mandated, the needle was kept as parallel as possible to the transducer to optimize continuous visualization of the needle shaft and tip. This also helped avoid placing the skin entry point too close to the transducer footprint and preserved a stable in-plane trajectory toward the target fascial plane.

Step 4: Confirmation of the Target Plane

Small-volume test injection is used to assess the spread pattern. Low-resistance spread with visible separation or “peeling” of the target fascial plane is considered suggestive of appropriate interfascial placement, whereas focal intramuscular resistance, pooling, or atypical spread should prompt reassessment of needle-tip position.

Step 5: Hydrodissection

In the clinical procedure, the painful side receives a total of 10 mL of injectate prepared to a final concentration of 15% dextrose with 0.1% lignocaine. The injectate is delivered incrementally under continuous ultrasound visualization, at an approximate rate of 10 mL per minute, with aspiration as clinically indicated and immediate adjustment if significant resistance, vascular flashback, or atypical spread is encountered.

Step 6: Endpoint

The procedural endpoint is visible longitudinal spread along the intended fascial plane at the distal TLF-gluteal junction. Operators should stop and reassess if the needle tip, spread pattern, or tissue response is not clearly visualized or appears atypical.

A practical procedural summary is provided in Table 2.

**Table 2:** Step-by-step workflow for ultrasound-guided distal thoracolumbar fascia (TLF) hydrodissection at the gluteal junction Practical procedural workflow, intended purpose, and key technical cues for ultrasound-guided distal thoracolumbar fascia hydrodissection at the gluteal junction.

Step	Maneuver	Practical purpose	Key endpoint or caution
1	Position patient prone	Optimize access to distal sacral-gluteal region	Use abdominal support if needed to reduce lordosis
2	Identify inferior sacroiliac joint margin, gluteus maximus, and distal TLF plane	Establish safe target below the sacroiliac joint	Maintain a clear sonographic map before needle insertion
3	Advance 22-gauge needle in plane from lateral to medial	Maintain continuous visualization and control	Avoid loss of tip visualization
4	Use small test injectate to assess plane	Verify interfascial rather than intramuscular placement	Look for low-resistance fascial separation; reassess if pooling or atypical spread occurs
5	Deliver total injectate incrementally	Achieve longitudinal hydrodissection along the target plane	Aspirate as indicated; stop if vascular flashback or atypical spread occurs
6	Observe spread pattern in real time	Assess procedural endpoint	Stop and reassess if tip, spread pattern, or tissue response is unclear or atypical

Clinical injectate safety considerations

The clinical injectate used the same total volume described above: 10 mL prepared to a final concentration of 15% dextrose with 0.1% lignocaine. No epinephrine was added. At a final lignocaine concentration of 0.1% and total injectate volume of 10 mL, the total lignocaine dose per procedure was 10 mg.

This formulation was used as a peri-fascial hydrodissection injectate rather than for direct intraneural, epidural, or intra-articular administration. This represented off-label peri-fascial use selected for hydrodissection and short-acting procedural analgesia. The inclusion of this formulation is intended for procedural context only; the present cadaveric technical report does not establish safety or efficacy for this injectate composition at the distal TLF target. Contraindications and precautions included local infection, inability to cooperate with positioning, known hypersensitivity or other relevant contraindication to any injectate component, coagulopathy or anticoagulant use requiring case-by-case assessment in accordance with local protocols and prescribing guidance, pregnancy where relevant, and any concern for progressive neurological deficit or serious underlying pathology requiring alternative evaluation. Standard safety measures included sterile preparation, real-time needle visualization, aspiration before each aliquot or as clinically indicated, post-procedure monitoring for 30 minutes, and immediate availability of resuscitation equipment and personnel trained to manage local anesthetic systemic toxicity and allergic reactions.

Cadaveric dye-spread assessment

Cadaveric injections used the same sonographic target and needle pathway as the clinical procedure. Each side received 10 mL of undiluted 1% methylene blue via a 22-gauge 3.5-inch spinal needle or a 22-gauge 4-inch hypodermic needle under continuous ultrasound guidance (Figure 3, Video 2). Undiluted 1% methylene blue was selected as a high-contrast gross tracer to facilitate macroscopic delineation of interfascial spread. The 10 mL injectate volume was chosen to reflect the intended clinical hydrodissection volume while allowing gross visualization of interfascial distribution without intentionally forcing deeper extension. All six injections were performed in plane from lateral to medial at the distal TLF-gluteal junction.

**Figure 3:**
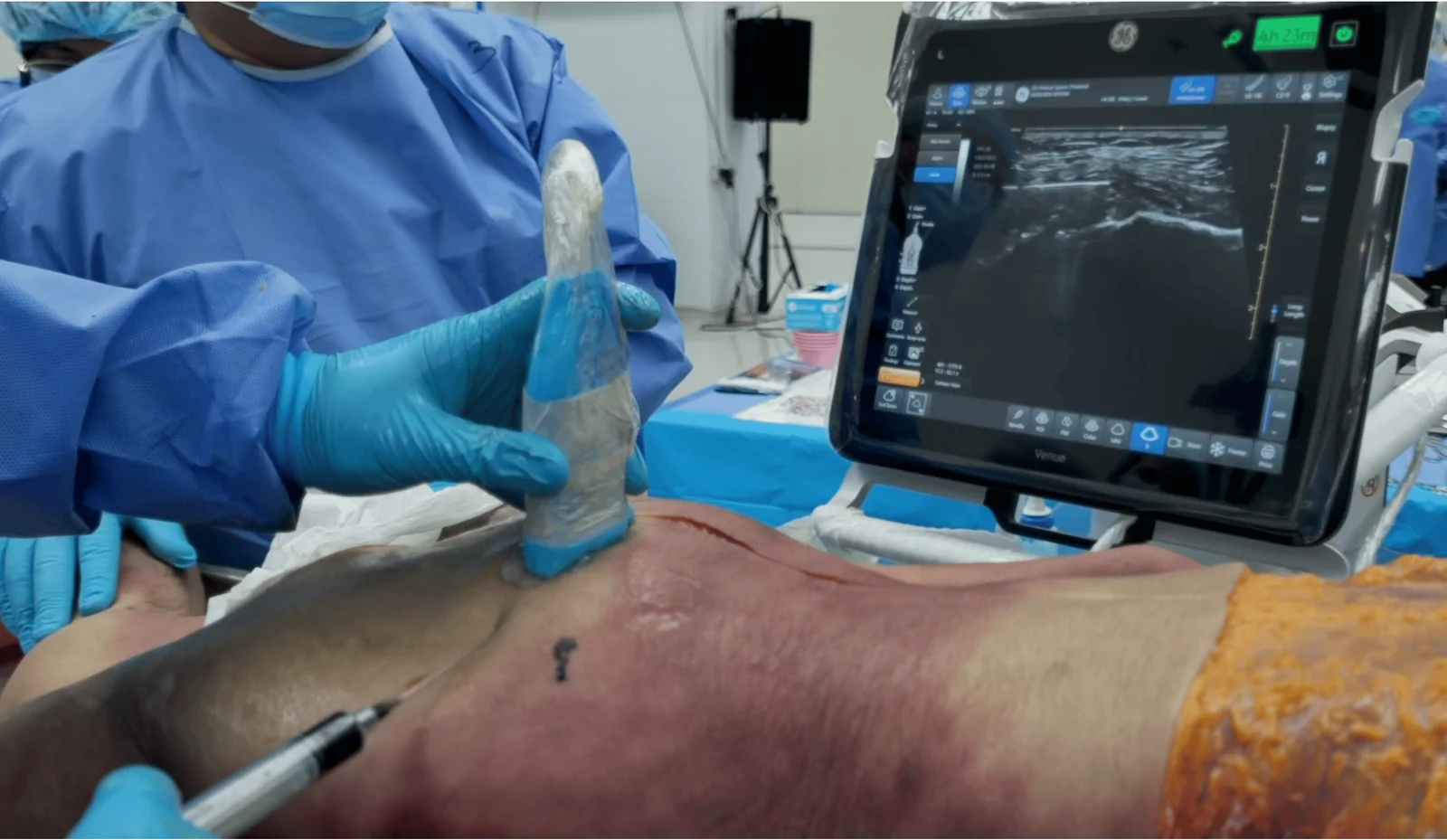
Ultrasound-guided setup for distal thoracolumbar fascia injection in the cadaveric model. Still image showing the procedural setup used for cadaveric methylene blue injection, including cadaver positioning, ultrasound machine orientation, transducer placement over the distal sacral-gluteal region, and in-plane needle trajectory toward the fascial plane deep to the gluteus maximus and superficial to the distal thoracolumbar fascia. The same ultrasound setup and needle approach were used in the clinical procedure. Image courtesy of Professor King Hei Stanley Lam.

**Video 2:** Ultrasound-guided cadaveric methylene blue injection at the distal thoracolumbar fascia. This video demonstrates ultrasound identification of the distal sacral and gluteal landmarks in the cadaveric model, followed by in-plane needle advancement to the target plane deep to the gluteus maximus and superficial to the distal thoracolumbar fascia, inferior to the sacroiliac joint. Methylene blue is then injected under real-time ultrasound guidance to reproduce the intended hydrodissection plane used in the clinical procedure. Video courtesy of Professor King Hei Stanley Lam.

The injected volume was delivered at an approximate rate of 10 mL per minute. Layered dissection was performed within a 15-30 minute post-injection workflow window. Exact side-specific intervals were not recorded in the original procedural log. Gross dissection proceeded in a standardized superficial-to-deep sequence to evaluate the gluteal fascia, gluteus maximus, distal TLF, sacrotuberous ligament, piriformis region, sciatic nerve, lumbosacral neural structures, and cranial extent of dye spread. Standardized photographic documentation was obtained at each major stage of layered dissection before further tissue reflection.

Gross staining was assessed by direct visualization during layered dissection. For each injected side, the presence or absence of visible methylene blue staining was recorded for predefined anatomical structures, including the deep surface of the gluteus maximus, TLF, multifidus, longissimus thoracis, iliocostalis lumborum, proximal sacrotuberous ligament, sciatic nerve, sacral plexus, and foraminal/epidural region. Cephalad spread to approximately the L3 level was assessed on each injected side using the same gross anatomical criteria, with sequential dissection referenced to lumbar spinous and transverse process landmarks. Approximate vertebral-level assignment was independently reviewed by a second examiner. Because bilateral injections were performed in each cadaver, the study design was descriptive and was not optimized to determine whether limited contralateral or midline crossover occurred.

Statistical analysis

Data were analyzed descriptively. Frequencies and proportions were used to summarize dye-distribution findings across injected sides. No inferential statistical analyses were performed because of the exploratory cadaveric design and very small sample size.

Cadaveric findings

Six injected sides from three fresh-frozen cadavers were included. Findings are reported descriptively by injected side. Across the six injected sides, methylene blue demonstrated cephalad spread to approximately the L3 level in all dissections (6/6, 100%). Multifidus and longissimus thoracis compartments were consistently stained (6/6, 100%), whereas no gross staining of the iliocostalis lumborum was observed in the injected sides examined (0/6, 0%). No gross dye was observed in the sciatic nerve, sacral plexus, neural foramina, or epidural space (0/6, 0%). No major specimen-level variability in the principal spread pattern was observed.

Cephalad dye spread to approximately the L3 level was observed on gross dissection in 6/6 injected sides (100%). Multifidus staining was present in 6/6 sides (100%), and longissimus thoracis staining was present in 6/6 sides (100%) (Figure 4A-4D). No gross staining of the iliocostalis lumborum was observed in 0/6 sides (0%). Partial staining of the proximal sacrotuberous ligament was observed in 6/6 sides (100%), and superficial staining of the adjacent piriformis region was seen where the gluteus maximus was reflected (Figure 4C). No gross dye was observed within the sciatic nerve, sacral plexus, neural foramina, or epidural space on dissection in 0/6 sides (0%). These findings are descriptive and should not be interpreted as excluding limited foraminal or epidural spread under other conditions. A schematic summary of the observed methylene blue spread pattern and staining extent in the transverse and coronal planes is provided in Figure 5A-5B.

**Figure 4:**
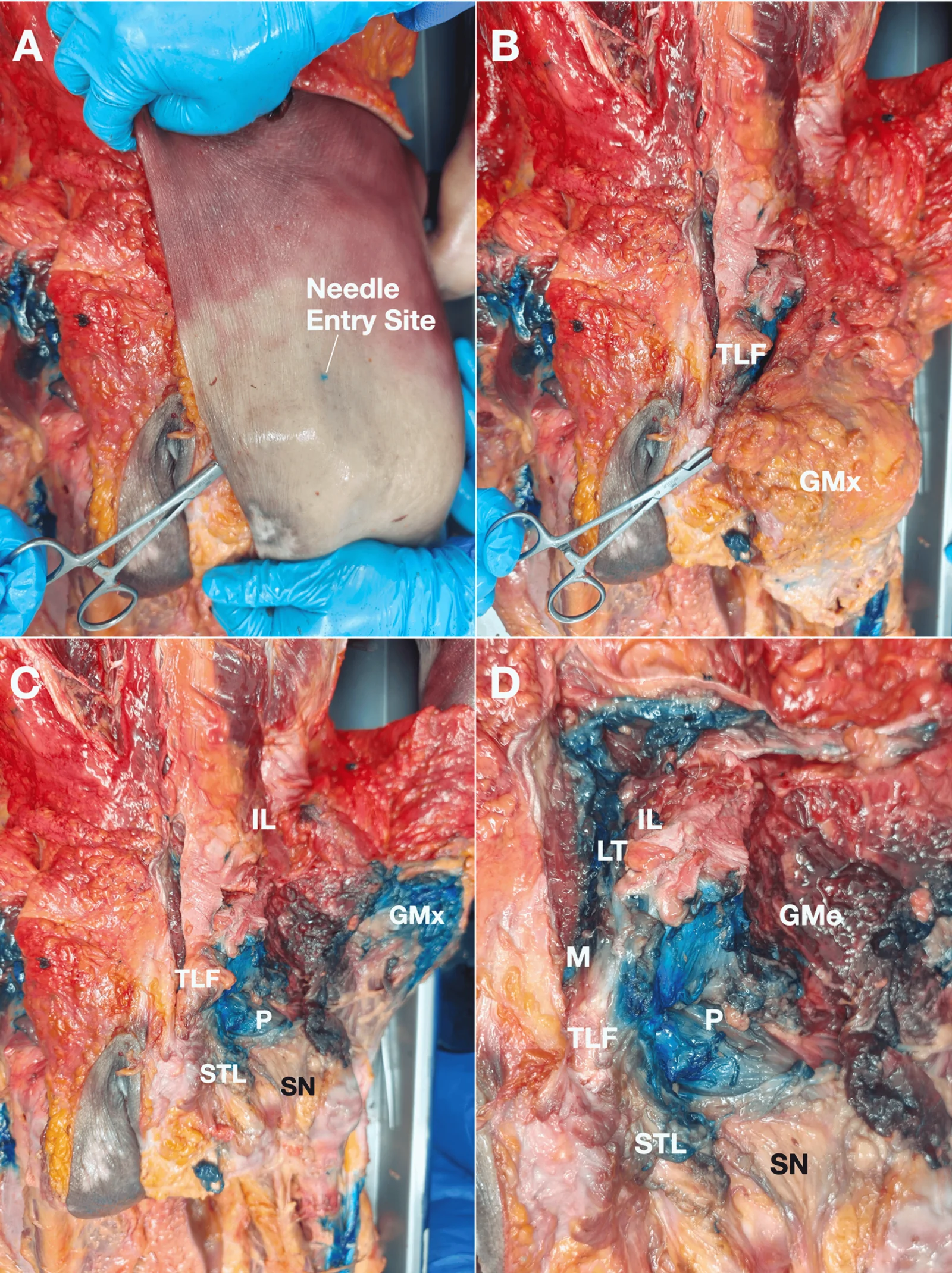
Layered cadaveric dissection demonstrating injectate distribution after ultrasound-guided distal thoracolumbar fascia hydrodissection. (A) Reconstructed external view of the cadaver after completion of dissection, with the skin and previously dissected tissues returned to their original position to demonstrate the needle entry point used for the injection. (B) Skin retracted to expose the gluteal fascia, gluteus maximus, and thoracolumbar fascia in the distal sacral-gluteal region. (C) Gluteus maximus retracted to expose its deep surface, demonstrating methylene blue staining of the thoracolumbar fascia, superficial fibers of the piriformis, and the proximal sacrotuberous ligament, with no gross sciatic nerve staining visible. (D) Thoracolumbar fascia dissected to reveal methylene blue staining of the multifidus and longissimus thoracis, with no gross staining of the iliocostalis lumborum, extending cranially to approximately the L3 level. Abbreviations: GF, gluteal fascia; GMe, gluteus medius; GMx, gluteus maximus; IL, iliocostalis lumborum; LT, longissimus thoracis; M, multifidus; P, piriformis; SN, sciatic nerve; STL, sacrotuberous ligament; TLF, thoracolumbar fascia. Image courtesy of Professor King Hei Stanley Lam.

**Figure 5:**
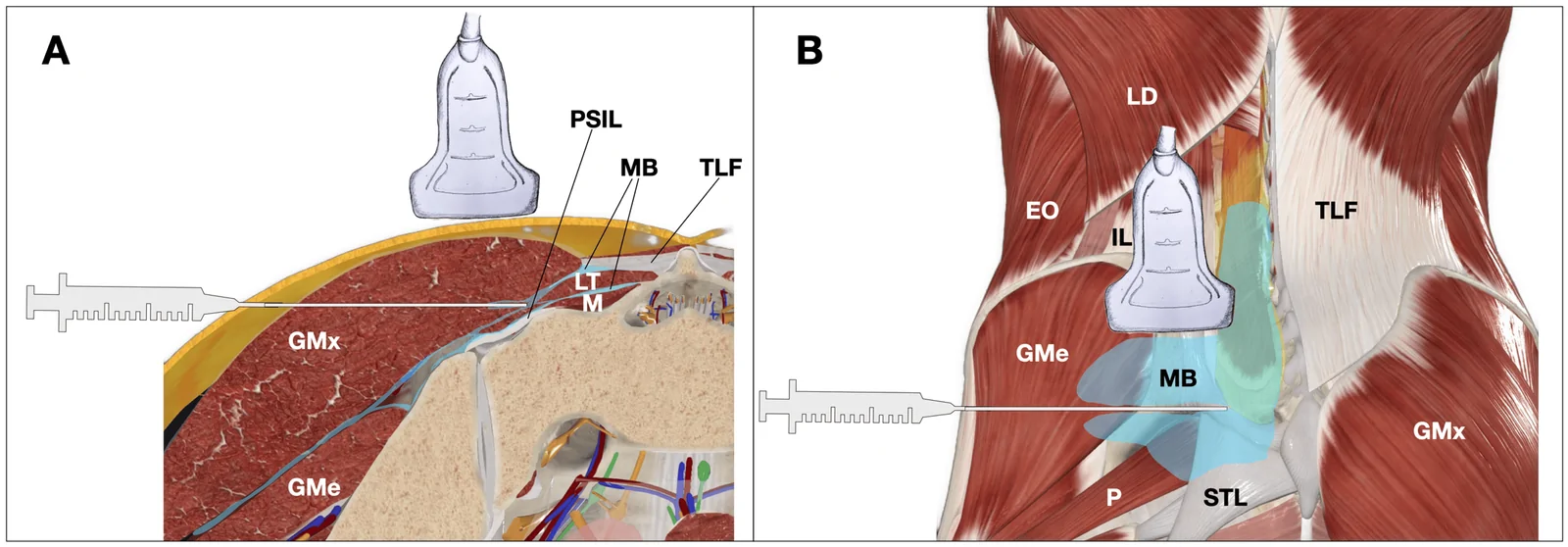
Schematic summary of methylene blue spread and staining extent after ultrasound-guided distal thoracolumbar fascia injection. (A) Transverse-plane schematic demonstrating preferential gross methylene blue (MB) distribution within the posterior fascial-paraspinal region, with staining of the multifidus and longissimus thoracis compartments and spread between the gluteus medius and gluteus maximus. (B) Coronal-plane schematic demonstrating cephalad spread to approximately the L3 level and inclusion of the proximal sacrotuberous ligament within the injected field in this cadaveric model. Preferential gross methylene blue (MB) distribution is shown within the posterior fascial-paraspinal region, with staining of the multifidus and longissimus thoracis compartments and no gross staining of the more lateral iliocostalis lumborum. The left gluteus maximus was removed to demonstrate MB spread, and the longissimus thoracis is faded to show the underlying multifidus. Abbreviations: GMe, gluteus medius; GMx, gluteus maximus; IL, iliocostalis lumborum; LT, longissimus thoracis; M, multifidus; MB, methylene blue; P, piriformis; PSIL, posterior sacroiliac ligament; STL, sacrotuberous ligament; TLF, thoracolumbar fascia. Credit: Schematic created in Keynote (Apple Inc., Cupertino, CA, USA) by Professor King Hei Stanley Lam.

Because both sides of each cadaver were injected, these findings should be interpreted as descriptive observations of observed compartmental spread and gross neural sparing rather than proof of strict unilateral confinement. The study design does not exclude limited contralateral or midline crossover.

A specimen-level summary is presented in Table 3.

**Table 3:** Summary of cadaveric dye-distribution findings by injected side Side-level descriptive summary of gross methylene blue distribution after bilateral ultrasound-guided distal thoracolumbar fascia injections in three fresh-frozen cadavers. Findings are reported descriptively by injected side; the three cadavers constituted the biological specimens.

Specimen	Deep gluteus maximus staining	TLF cephalad spread	Multifidus staining	Longissimus staining	Iliocostalis staining	Proximal sacrotuberous ligament staining	Sciatic nerve staining	Sacral plexus staining	Foraminal/epidural spread
Cadaver 1, right	Present	Present to ~L3	Present	Present	Absent	Present, partial	Absent	Absent	Absent
Cadaver 1, left	Present	Present to ~L3	Present	Present	Absent	Present, partial	Absent	Absent	Absent
Cadaver 2, right	Present	Present to ~L3	Present	Present	Absent	Present, partial	Absent	Absent	Absent
Cadaver 2, left	Present	Present to ~L3	Present	Present	Absent	Present, partial	Absent	Absent	Absent
Cadaver 3, right	Present	Present to ~L3	Present	Present	Absent	Present, partial	Absent	Absent	Absent
Cadaver 3, left	Present	Present to ~L3	Present	Present	Absent	Present, partial	Absent	Absent	Absent
Overall frequency	6/6 (100%)	6/6 (100%)	6/6 (100%)	6/6 (100%)	0/6 (0%)	6/6 (100%)	0/6 (0%)	0/6 (0%)	0/6 (0%)

Technical pearls

Several practical points were consistently helpful. First, targeting the distal TLF at the gluteal junction worked best when the inferior border of the sacroiliac joint was identified before needle insertion, as this reduced the risk of drifting too cranially or too medially. Second, low-resistance spread with visible fascial separation was a useful ultrasound sign suggestive of appropriate interfascial placement, whereas higher resistance, focal pooling, or atypical spread prompted reassessment. Third, continuous in-plane tip visualization was especially important because the target lies deep to the gluteus maximus and near important neurovascular structures. Fourth, longitudinal spread along the intended fascial plane was more informative than focal pooling at a single point.

Safety considerations

This report describes gross anatomical findings after cadaveric injection, not proven biological or clinical safety. On layered dissection, no gross dye was observed in the sciatic nerve, sacral plexus, neural foramina, or epidural space in the injected sides examined, supporting gross neural sparing under the tested cadaveric conditions. However, absence of gross neural staining does not exclude microscopic, intravascular, or deeper spread, does not exclude limited foraminal or epidural spread under other conditions, and does not establish histologic or in vivo safety.

Procedurally, safety depends on maintaining a clearly visualized in-plane needle path, remaining inferior to the sacroiliac joint, avoiding unnecessary medial depth, and stopping immediately if vascular flashback, marked resistance, or atypical spread is encountered. All clinical use should include standard contraindication screening, aspiration practices, real-time monitoring, and readiness to manage adverse reactions to lignocaine or vasovagal events. The cadaveric model cannot account for living tissue pressure, vascular uptake, pain provocation, dynamic tissue compliance, or delayed complications. The present cadaveric findings should not be interpreted as evidence supporting the safety or efficacy of the clinical injectate formulation at this target in living patients.

## Discussion

This technical report describes an ultrasound-guided distal TLF hydrodissection technique at the gluteal junction and presents cadaveric evidence of an observed gross dye-spread pattern within the posterior fascial-paraspinal region. In all six injected sides, dye tracked cranially to approximately the L3 level and involved the multifidus and longissimus thoracis compartments, while no gross staining of the iliocostalis lumborum was observed. Partial proximal sacrotuberous ligament staining was also observed. No gross dye was observed in the sciatic nerve, sacral plexus, neural foramina, or epidural space in the injected sides examined, although these findings should not be interpreted as excluding limited spread under other conditions.

These findings are anatomically plausible and broadly consistent with prior descriptions of thoracolumbar fascia continuity and compartmental organization in the posterior trunk [1-3,7]. The observed dye pattern supports the concept that a caudal gluteal entry point can provide access to a posterior fascial-paraspinal region without direct foraminal or epidural targeting. The mechanism by which dye introduced at a superficial interfascial plane reached the deeper multifidus and longissimus thoracis compartments was not determined in this study; possible contributors include regional fascial continuity, injection pressure, and cadaveric tissue compliance. At the same time, the present study does not establish a discrete universal anatomical channel, nor does it determine clinical relevance of the stained compartments as pain generators. Rather, it provides an anatomical and technical observation that may inform future in vivo investigation.

The consistent staining of multifidus and longissimus thoracis, together with absence of gross iliocostalis staining in the injected sides examined, suggests that gross dye spread in this cadaveric model was not indiscriminate throughout the posterior trunk. The observed gross dye pattern was consistent with preferential distribution within a more specific interfascial region, although confinement cannot be established by the present design. From a technical standpoint, the distal gluteal-TLF junction may provide a practical sonographic route to the posterior fascial-paraspinal region observed in this cadaveric model. The clinical significance of targeting this region requires dedicated in vivo study.

The observed partial staining of the proximal sacrotuberous ligament is also notable. This finding indicates that the injected field may include the proximal sacrotuberous ligament in this cadaveric model. The clinical implications of this observed distribution were not evaluated in the present study and require dedicated in vivo investigation.

The main contribution of this report is therefore technical and anatomical rather than therapeutic. It demonstrates that, in a fresh-frozen cadaver model, ultrasound-guided injection at the distal thoracolumbar fascia-gluteal junction was technically achievable and produced a reproducible gross dye-spread pattern under the conditions tested. The study does not establish clinical effectiveness, does not establish safety or efficacy of the described clinical injectate formulation at this target, and does not test whether the targeted region is a validated source of symptoms.

Several limitations warrant emphasis. First, the study was cadaveric and descriptive, with only three older cadavers (mean age 74.3 years), which limits generalizability to younger individuals or patients with different fascial morphology. Second, fresh-frozen tissue does not reproduce living perfusion, tissue compliance, pain feedback, respiration, or fascial glide. Third, injectate pressure was not measured. Fourth, no histologic confirmation or post-injection imaging confirmation was performed. Fifth, formal interobserver agreement statistics were not calculated. Sixth, the injection-to-dissection interval was not identical across all sides and was recorded only as a 15-30 minute workflow window, which may have influenced the extent of gross dye spread. Finally, because both sides of each cadaver were injected, the study was not designed to assess strict unilateral confinement or subtle contralateral crossover.

Prospective clinical studies, ideally with standardized imaging correlation and carefully reported safety monitoring, will be necessary before any conclusions about clinical effectiveness or comparative value can be made. Future clinical evaluation should also consider whether practitioners can obtain the imaging needed for appropriate patient assessment and follow-up [16].

## Conclusions

In this fresh-frozen cadaver model, ultrasound-guided injection at the distal thoracolumbar fascia-gluteal junction was technically achievable and produced gross cephalad dye spread to approximately the L3 level. Multifidus and longissimus thoracis staining were observed in all injected sides, no gross iliocostalis staining was observed in the injected sides examined, and partial proximal sacrotuberous ligament staining was present. No gross dye was observed in the sciatic nerve, sacral plexus, neural foramina, or epidural space in the injected sides examined.

These findings describe an observed pattern of cadaveric compartmental dye spread with gross neural sparing under the tested conditions. They do not establish clinical effectiveness, do not exclude microscopic spread, and do not establish histologic or in vivo safety. This report also does not establish safety or efficacy of the described injectate formulation at the targeted distal thoracolumbar fascia plane. Accordingly, this cadaveric technical report should be interpreted as hypothesis-generating rather than evidence of clinical effectiveness or safety. Future prospective clinical studies should define patient selection criteria clearly, collect standardized pain and functional outcome measures, document adverse events systematically, and use imaging or other appropriate methods to assess in vivo injectate distribution.
